# High‐dose Methotrexate plus temozolomide with or without rituximab in patients with untreated primary central nervous system lymphoma: A retrospective study from China

**DOI:** 10.1002/cam4.1906

**Published:** 2019-03-01

**Authors:** Cui Chen, Peng Sun, Juan Cui, Shumei Yan, Hao Chen, Yi Xia, Xiwen Bi, Panpan Liu, Yu Wang, Hang Yang, Man Nie, Xue‐Wen Zhang, Wenqi Jiang, Zhi‐Ming Li

**Affiliations:** ^1^ Department of Medical Oncology Sun Yat‐Sen University Cancer Center Guangzhou China; ^2^ State Key Laboratory of Oncology in South China Collaborative Innovation Center for Cancer Medicine Guangzhou China; ^3^ Department of Oncology, the First Affiliated Hospital Sun Yat‐Sen University Guangzhou China; ^4^ Ward One, Department of Chemotherapy Wuzhou Red Cross Hospital Wuzhou China; ^5^ Department of Pathology Sun Yat‐Sen University Cancer Center Guangzhou China; ^6^ Department of Clinical Laboratory Medicine Sun Yat‐Sen University Cancer Center Guangzhou China

**Keywords:** chemotherapy, high‐dose methotrexate (HD‐MTX), primary central nervous system lymphoma (PCNSL), rituximab, temozolomide (TMZ)

## Abstract

The purpose of this retrospective study was to compare the efficacy and toxicity of high‐dose methotrexate plus temozolomide (MT regimen) and rituximab plus MT (RMT regimen) in patients with untreated primary central nervous system lymphoma (PCNSL). A total of 62 patients with untreated PCNSL were enrolled between January 2005 and December 2015, with the median age of 53.5 years (range 29‐77).In this study, 32 patients received RMT as induction therapy, and 30 received MT. Objective responses were noted in 93.7% of the patients in the RMT group and in 69.0% of the patients in the MT group (*P *= 0.018), while complete responses were noted in 53.2% of the patients in the RMT group and 27.6% of the patients in the MT group (*P *< 0.001). The 2‐ and 5‐year PFS rates were 81.3% and 53.3%, respectively, for the RMT group and 46.5% and 29.1%, respectively, for the MT group (*P *= 0.019). The 2‐ and 5‐year overall survival (OS) rates were 82.3% and 82.3%, respectively, for the RMT group and 65.7% and 50.0%, respectively, for the MT group (*P *= 0.015). Multivariate analyses showed that therapeutic regimen (RMT vs MT) was an independent prognostic factor for PFS and OS. Our encouraging results suggest that the RMT regimen may be a feasible and safe therapeutic approach for first‐line treatment of PCNSL.

## Abstract 2

The purpose of this study was to compare the efficacy and toxicity of high‐dose methotrexate plus temozolomide (MT regimen) and of rituximab plus MT (RMT regimen) in untreated PCNSL. Our encouraging results suggest that the RMT regimen might be a feasible and safe therapeutic approach in the first‐line treatment of PCNSL.

## 1 INTRODUCTION

Primary central nervous system lymphoma (PCNSL) is a rare type of extranodal lymphoma and is confined exclusively to the central nervous system (CNS), accounting for approximately 2%‐3% of primary brain malignancies.^1^, ^2^ Due to its distinct site of occurrence and aggressive biological behavior, PCNSL has an unsatisfactory clinical outcome.^3^

Whole‐brain radiation therapy (WBRT) was regarded as the frontline treatment for PCNSL until the 1990s and achieved a median overall survival (OS) of 12‐17 months.^4^, ^5^ Because combined chemotherapy significantly increased the response rate and survival of patients with systematic lymphomas, a variety of chemotherapy agents and regimens were also explored for PCNSL. High‐dose methotrexate (HD‐MTX) was then proven to reach a therapeutic concentration in the brain and was found to improve survival when added to WBRT.^6^, ^7^ Subsequently, many drugs in combination with HD‐MTX have been investigated to improve the response rate and to prolong the survival of patients with PCNSL. The International Extranodal Lymphoma Study Group (IELSG) 20 demonstrated that compared with HD‐MTX monotherapy, high‐dose cytarabine (Ara‐C) combined with HD‐MTX (MA regimen) significantly improved the complete response rate and progression‐free survival (PFS) of patients with PCNSL.^8^ Based on this finding, the MA regimen has been regarded as one of the standard approaches for PCNSL.

However, the MA regimen has a high incidence of severe toxicities, which are not well tolerated in weak or elderly patients.^8^ Therefore, low‐toxicity therapeutics were further investigated for PCNSL to balance intensification of therapy with regulation of side effects. Temozolomide (TMZ) is an oral alkylating agent that can penetrate the blood‐brain barrier (BBB) and achieve a high concentration in the CNS.^9^, ^10^ TMZ is traditionally used to treat glioma and has reported activity in PCNSL.^9^, ^10^, ^11^, ^12^, ^13^, ^14^, ^15^, ^16^ The combination of HD‐MTX and TMZ (MT) has also shown comparable effects and has achieved an acceptable survival rate for PCNSL patients, with an objective response rate (ORR) of approximately 70%‐80% and a 2‐year OS rate of 39%‐62%.^10^, ^16^ More importantly, the MT regimen has relatively low toxicities and is well tolerated in elderly and physically weak patients.^10^, ^17^

Rituximab is a chimeric anti‐CD20 monoclonal antibody and is widely used for the treatment of CD20+ non‐Hodgkin lymphoma.^18^ Because 90%‐95% of PCNSLs are pathologically diagnosed as diffuse large B‐cell lymphoma (DLBCL), rituximab can theoretically enhance the efficacy of chemotherapy in PCNSL.^19^ An increasing number of studies and meta‐analyses have investigated the effect of rituximab in PCNSL, indicating that rituximab can robustly enhance the response rate and possibly improve survival.^20^, ^21^, ^22^, ^23^ Thus, we hypothesized that rituximab may potentiate the effectiveness of MT in patients with PCNSL as an initial treatment. However, data regarding the addition of rituximab to MT (RMT regimen) for PCNSL are limited, and no study has directly compared the efficacy of RMT to that of MT. To address this problem, we analyzed and compared the efficacy and safety of RMT and MT in untreated PCNSL patients from Southern China.

## 2 PATIENTS AND METHODS

### 2.1 Patients

All patients diagnosed with PCNSL between January 2005 and December 2015 were retrospectively reviewed. The included patients met the following criteria: (a) the disease was pathologically diagnosed as DLBCL; (b) complete clinical and treatment information were available; (c) the patients were between 18 and 80 years of age; (d)there was no involvement of sites other than the CNS; (e) no antitumor treatment was received before admission; and (f) the patient presented with at least one measurable lesion. The exclusion criteria were (a) patients with other types of malignancy and (b) patients with any immunodeficiency disease. Finally, a total of 62 patients were enrolled.

### 2.2 Treatment

MT regimen: Methotrexate (MTX) (3.5 g/m^2^) was intravenously administered on Day 1, and TMZ (150 mg/m^2^) was orally administered on Days 1‐5, with or without rituximab (375 mg/m^2^), which was intravenously administered on Day 0. The regimen was repeated every 3 weeks. Adequate hydration was provided. Each dose of MTX was followed 12 hours later by leucovorin 30 mg every 6 hours. MTX levels were measured every 12 hours. Leucovorin was stopped when the MTX level was <1×10^−7 ^mol/L. In our center, physicians should determine the appropriate first‐line regimen according to the each patient's disease condition and economic status. The patients received up to 6‐8 cycles of induction therapy. Chemotherapy was discontinued if the disease progressed or if intolerable toxicity developed. According to the physicians' decisions and the patients' willingness, patients received autologous stem‐cell transplantation (ASCT) or WBRT as consolidation therapy.

### 2.3 Treatment evaluation and toxicity

Treatment responses were assessed by contrast‐enhanced magnetic resonance imaging (MRI) of the brain, which was performed at baseline and after the second, fourth, and sixth cycles of chemotherapy. Complete remission (CR) was defined as complete disappearance of all lesions, a partial response (PR) was defined as a ≥50% decrease in the size of the enhancing tumor, progressive disease (PD) was defined as a ≥25% increase in tumor size or the occurrence of a new lesion, and stable disease (SD)was defined as a situation that could not be classified as CR, PR, or PD. After completing treatment, the patients were evaluated by repeat contrast‐enhanced MRI of the brain every 3 months for the first 2 years and then every 6 months for years 3‐5. Upon cessation of treatment, each patient was followed up every 3 months at the clinic or by telephone interview until 5 years. Treatment‐related adverse events were evaluated with the Common Terminology Criteria for Adverse Events (CTCAE) version 3.0.^24^

### 2.4 Statistical analyses

The patient characteristics and treatment responses of the two therapeutic groups (RMT vs MT) were compared using the chi‐square or Fisher’s exact tests. Survival was estimated with the Kaplan–Meier method and compared using the log‐rank test. OS was defined as the time from the date of diagnosis to the date of death or the last follow‐up visit, and PFS was defined as the time from diagnosis to relapse, progression, death or the date of the last follow‐up visit. Univariate and multivariate survival analyses were performed based on the Cox proportional hazards regression methodology. Hazard ratios (HRs) with 95% CIs and two‐sided *P* values were reported. An alpha value of *P* < 0.05 was considered statistically significant. The statistical analyses were performed using the Statistical Package for the Social Sciences version 22.0.

## 3 RESULTS

In this study, 32 patients received RMT as induction therapy, and 30 patients received MT. The median age of the entire cohort was 53.5 years (range 29‐77). Thirty‐two male patients and 30 female patients were included, with a sex ratio of 1.07. Among them, 20 patients (32.3%) received WBRT as consolidation therapy, while only 2 patients (3.2%) received ASCT. Two previously reported prognostic models for PCNSL, namely, the IESLG model^25^ and MSKCC model,^26^ were also introduced to stratify the risk groups of our patients. As listed in Table 1, except for gender, the clinical characteristics were generally well balanced between the two treatment groups (RMT vs MT). A considerably higher percentage of male patients was present in the RMT group than in MT group (65.6% vs 36.7%, *P *= 0.041).

**Table 1:** Baseline clinical characteristics of 62 patients with untreated PCNSL

Characteristic	Total (%)	RMT (%)	MT (%)	*P* value
Gender				
Male	32 (51.6)	21 (65.6)	11 (36.7)	0.041^*^
Female	30 (48.4)	11 (34.4)	19 (63.3)	
Age (y)				
Median (Range)	53.5 (29‐77)	55 (29‐77)	53 (30‐72)	
≤60	41 (66.1)	19 (59.4)	22 (73.3)	0.291
＞60	21 (33.9)	13 (40.6)	8 (26.7)	
Performance status				
KPS≥90	22 (35.5)	11 (34.4)	11 (36.7)	1.000
KPS＜90	40 (64.5)	21 (65.6)	19 (63.3)	
IELSG model				
Low (0‐1)	26 (41.9)	12 (37.5)	14 (46.7)	0.331
Intermediate (2‐3)	34 (54.8)	18 (56.3)	16 (53.3)	
High (4‐5)	2 (3.2)	2 (6.2)	0 (0)	
MSKCC model				
Low	21 (33.9)	8 (25.0)	13 (43.3)	0.115
Intermediate	37 (59.7)	23 (71.9)	14 (46.7)	
High	4 (6.5)	1 (3.1)	3 (10.0)	
Multiple lesions	34 (56.7)	14 (45.2)	20 (69.0)	0.074
LDH elevated	1 (1.6)	1 (3.1)	0 (0)	1.000
Positive CSF cytology	13 (21.0)	7 (21.9)	6 (20.0)	1.000
Deep structure involvement	21 (35.6)	12 (40.0)	9 (31.0)	0.589
WBRT	20 (32.3)	12 (37.5)	8 (26.7)	0.423
ASCT	2 (3.2)	2 (6.3)	0 (0)	0.492

ASCT, autologous stem‐cell transplantation; CSF, cerebro‐spinal fluid; IELSG, International Extranodal Lymphoma Study Group; LDH, lactate dehydrogenase; MSKCC, Memorial Sloan Kettering Cancer Center; MT, combination regimen of high‐dose methotrexate and temozolomide; RMT, combination regimen of rituximab, high‐dose methotrexate and temozolomide; WBRT, whole‐brain radiation therapy.

The response rates are shown in Table 2. Sixty‐one patients were evaluated for responses. CR was observed in 17 patients (53.2%) on RMT and 8 patients (27.6%) on MT (*P *< 0.001), while PR was observed in 13 patients (40.6%) on RMT and 12 patients (41.4%) on MT (*P *= 0.572). The RMT regimen achieved a significantly higher ORR than the MT regimen (93.7% vs 69.0%, *P *= 0.018).

**Table 2:** Evaluation of treatment response

Treatment response	Total (%)	RMT (%)	MT (%)	*P* value
CR+PR	50 (82.0)	30 (93.7)	20 (69.0)	0.018*
SD+PD	11 (18.0)	2 (6.3)	9 (31.0)	
CR	25 (41.0)	17 (53.2)	8 (27.6)	＜0.001*
PR	25 (41.0)	13 (40.6)	12 (41.4)	0.572
PD	8 (13.1)	1 (3.1)	7 (24.1)	＜0.001*
SD	3 (4.9)	1 (3.1)	2 (6.9)	0.296

CR, complete remission; MT, combination regimen of high‐dose methotrexate and temozolomide; PD, progressive disease; PR, partial remission; RMT, combination regimen of rituximab, high‐dose methotrexate and temozolomide; SD, stable disease.

* *P* < 0.05

No toxic death was observed. Febrile neutropenia (FN) occurred in three patients (9.4%) on RMT and in two patients (6.7%) on MT (*P *= 1.000). Grade 3‐4 hematological toxicities (anemia, neutropenia, and thrombocytopenia) were not frequent in either group (RMT vs MT), with no significant difference between them (all *P *> 0.05). Grade 1‐2 hepatotoxicity was observed in 13 patients (40.6%) on RMT and in 12 patients (40%) on MT (*P *= 1.000), and grade 1‐2 nausea/vomiting was observed in 14 patients (46.6%) on RMT and in 18 patients (62.5%) on MT (*P *= 0.049). Grade 3‐4 non‐hematological toxicities were generally uncommon in both groups. All toxicity data are summarized in Table 3.

**Table 3:** Grade 3‐4 toxicities

Toxicities	RMT (%)	MT (%)	*P* value
Neutropenia	9 (28.1)	8 (26.7)	1.000
Thrombocytopenia	4 (12.5)	3 (10)	1.000
Anemia	7 (21.9)	3 (10)	0.304
Hematological toxicity	11 (34.4)	11 (36.7)	1.000
Febrile neutropenia	3 (9.4)	2 (6.7)	1.000
Nausea/vomiting	0 (0)	2 (6.7)	0.230
Mucositis	0	0	—
Pneumonia	4 (12.5)	2 (6.7)	0.672
Hepatotoxicity	1 (3.1)	1 (3.3)	1.000
Nephrotoxicity	0	0	—
Cardiotoxicity	0	1 (3.3)	0.484
Neurotoxicity	0	0	—
Toxic deaths	0	0	—

MT, combination regimen of high‐dose methotrexate and temozolomide; RMT, combination regimen of rituximab, high‐dose methotrexate and temozolomide.

**Figure 1:**
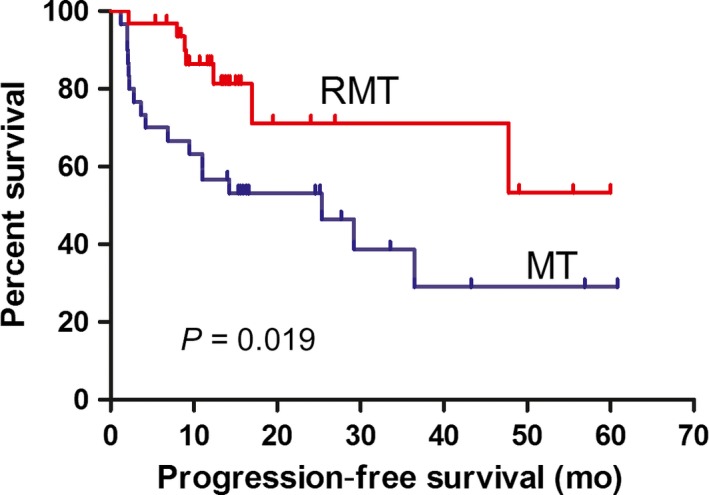
Kaplan–Meier curves for progression‐free survival (PFS) with MT and RMT

**Figure 2:**
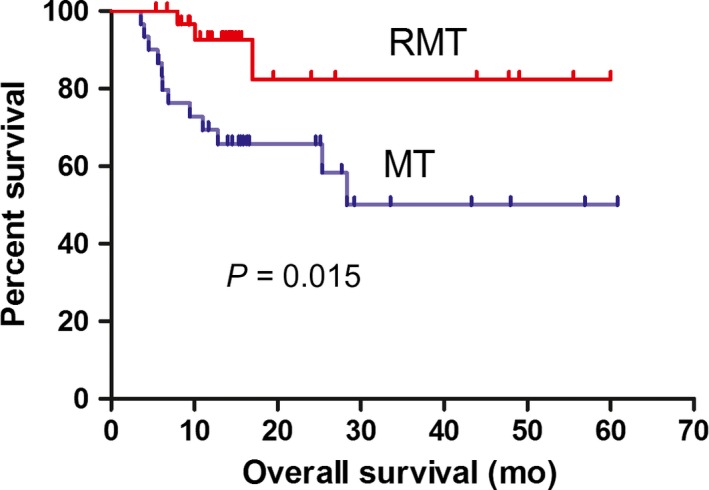
Kaplan–Meier curves for overall survival (OS) with MT and RMT

Over a median follow‐up time of 14.2 months (3.57‐60.8 months), 15 deaths were observed (12 patients on MT and 3 patients on RMT), all of which were due to tumor progression or relapse. The median follow‐up time was 15.5 months for the MT group and 13.7 months for the RMT group. Six patients in the RMT group and 10 patients in the MT group relapsed, and none of them showed extra‐CNS involvement. The 2‐ and 5‐year PFS rates were 81.3% and 53.3%, respectively, for the RMT group and 46.5% and 29.1%, respectively, for the MT group (*P *= 0.019). The 2‐ and 5‐year OS rates were 82.3% and 82.3%, respectively, for the RMT group and 65.7% and 50.0%, respectively, for the MT group (*P *= 0.015). The median PFS time was 25.3 months in the MT group and was not reached in the RMT group. The median OS time was not reached in either group. Kaplan–Meier survival curves of the PFS and OS were constructed (Figure 1 and 2).

To further determine the prognostic impacts of the therapeutic approaches on PCNSL, a Cox regression model was generated. Deep structure involvement and treatment approach were identified as prognostic factors for PFS in the univariate analysis, and they were both proven to be independent prognostic factors of PFS in the multivariate analysis after adjusting for gender and age (Table 4). Univariate analysis demonstrated that treatment approach was the only prognostic factor for OS. After adjusting for age and gender, treatment approach was identified as an independent prognostic factor for OS in the multivariate analysis (Table 5). Compared with the MT regimen, the RMT regimen reduced the risk of progression by 75% and the risk of mortality by 81.9% in PCNSL. Unfortunately, neither the IELSG model nor the MSKCC model was found to correlate with PFS or OS in the survival analyses.

**Table 4:** Univariate and multivariate analysis of progression‐free survival (PFS)

Variable	Univariate				Multivariate			
	*P* value	HR	95% CI		*P* value	HR	95% CI	
			Lower	Upper			Lower	Upper
Gender								
Male	Reference				Reference			
Female	0.815	0.909	0.407	2.030	0.652	0.799	0.301	2.119
Age (y)								
≤60	Reference				Reference			
＞60	0.332	0.646	0.267	1.563	0.835	0.898	0.328	2.462
Performance status								
KPS≥90	Reference							
KPS＜90	0.632	0.819	0.363	1.849				
IELSG model								
Low (0‐1)	Reference							
Intermediate‐High (2‐5)	0.263	0.632	0.283	1.410				
MSKCC model								
Low	Reference							
Intermediate‐High	0.164	0.563	0.251	1.264				
Multiple lesions								
Absent	Reference							
Present	0.768	0.884	0.389	2.009				
Positive CSF cytology								
Absent	Reference							
Present	0.064	2.317	0.952	5.637				
Deep structure involvement								
Absent	Reference				Reference			
Present	0.025*	0.248	0.073	0.841	0.030*	0.243	0.068	0.870
WBRT								
Absent	Reference							
Present	0.153	0.519	0.211	1.277				
Regimen								
MT	Reference				Reference			
RMT	0.019*	0.361	0.149	0.876	0.018*	0.235	0.071	0.782

CI, confidential interval; CSF, cerebro‐spinal fluid; HR, hazard ratio; IELSG, International Extranodal Lymphoma Study Group; MSKCC, Memorial Sloan Kettering Cancer Center; MT, combination regimen of high‐dose methotrexate and temozolomide; PFS, progression‐free survival; RMT, combination regimen of rituximab, high‐dose methotrexate and temozolomide; WBRT, whole‐brain radiation therapy.

* *P *< 0.05

**Table 5:** Univariate and multivariate analysis of overall survival (OS)

Variable	Univariate				Multivariate			
	*P* value	HR	95% CI		*P* value	HR	95% CI	
			Lower	Upper			Lower	Upper
Gender								
Male	Reference				Reference			
Female	0.566	1.354	0.481	3.813	0.531	0.696	0.224	2.166
Age (y)								
≤60	Reference				Reference			
＞60	0.798	1.145	0.406	3.228	0.344	1.693	0.569	5.037
Performance status								
KPS≥90	Reference							
KPS＜90	0.727	1.211	0.413	3.555				
IELSG model								
Low (0‐1)	Reference							
Intermediate‐High (2‐5)	0.548	0.732	0.264	2.027				
MSKCC model								
Low	Reference							
Intermediate‐High	0.519	0.711	0.252	2.008				
Multiple lesions								
Absent	Reference							
Present	0.891	1.076	0.378	3.057				
Positive CSF cytology								
Absent	Reference							
Present	0.110	2.414	0.820	7.108				
Deep structure involvement								
Absent	Reference							
Present	0.065	0.246	0.056	1.093				
WBRT								
Absent	Reference							
Present	0.090	0.330	0.092	1.189				
Regimen								
MT	Reference				Reference			
RMT	0.015*	0.234	0.066	0.833	0.016*	0.181	0.045	0.726

CI, confidential interval; CSF, cerebro‐spinal fluid; HR, hazard ratio; IELSG, International Extranodal Lymphoma Study Group; MSKCC, Memorial Sloan Kettering Cancer Center; MT, combination regimen of high‐dose methotrexate and temozolomide; OS, overall survival; RMT, combination regimen of rituximab, high‐dose methotrexate and temozolomide; WBRT, whole‐brain radiation therapy

* *P *< 0.05

## 4 DISCUSSION

To the best of our knowledge, this is the first study to investigate the MT regimen and RMT regimen in untreated PCNSL patients. In the current study, both the MT regimen and the RMT regimen yielded favorable clinical outcomes in PCNSL and were well tolerated. Notably, the addition of rituximab significantly improved the response rate (ORR 93.7% and CRR 53.2%) and survival rate in the RMT group. Furthermore, the data suggested that rituximab can provide an additional benefit when added to conventional HD‐MTX‐based polychemotherapy.

For decades, the HD‐MTX‐combined regimen has been regarded as the cornerstone of chemotherapy in PCNSL,^7^, ^27^ and a variety of agents have been investigated in combination with HD‐MTX for PCNSL. The first randomized trial assessing chemotherapy in PCNSL was reported in 2009. In that phase 2 prospective study, high‐dose Ara‐C significantly improved the clinical outcome of PCNSL when added to HD‐MTX.^8^ However, 92% of patients in the MA group developed grade 3‐4 hematological toxicities, and dose reductions and therapy discontinuation were frequent.^8^ Consequently, the frequent severe toxicities limited the widespread use of MA in PCNSL, especially in frail populations. Subsequently, several alkylating agents in combination with HD‐MTX without cytarabine were successfully used in the first‐line care of PCNSL worldwide.^10^, ^28^, ^29^, ^30^, ^31^, ^32^ Among them, TMZ was found to be a promising agent for combination with HD‐MTX. In the retrospective series reported by Omuro et al^13^ and Wang et al,^17^ the authors demonstrated that the effect of MT was comparable to that of MA for newly diagnosed PCNSL patients, including elderly patients. The ANOCEF‐GOELAMS trial provided data regarding the MT regimen in elderly PCNSL patients, with 48 patients randomized into the MT group.^10^ The MT regimen achieved an ORR of 71% but yielded a median OS of 14 months, with tolerable toxicity.^10^ Consistently, our study showed a similar ORR (69%) for MT to that reported in previous studies. Most importantly, the toxicity of MT was mild in all reported studies and could be well tolerated by elderly patients.

Rituximab, a CD20 antibody, has greatly advanced DLBCL therapy in the past two decades and is widely indicated for the treatment of other CD20‐positive non‐Hodgkin lymphomas.^18^ Because most PCNSLs are CD20+ and may therefore be responsive to rituximab,^19^ further exploration of the treatment effect of rituximab in PCNSL is warranted. Gregory et al conducted a retrospective analysis of patients with PCNSL and noted that rituximab improved outcomes when added to methotrexate.^23^ Unlike our study, patients diagnosed before 2004 were also included, and only a few patients received rituximab (18%, including a non‐RMT regimen) in Gregory's study. In addition, the patients in Gregory's study received various chemotherapeutic regimens and the dose of MTX was not uniform. These factors may have rendered Gregory's study underpowered to identify statistical significance for rituximab in a multivariate analysis of survival. In a phase 2 trial (CALGB 50202) by Rubenstein et al,^33^ patients received a combination regimen of HD‐MTX (8g/m^2^), TMZ and rituximab, and the patients who achieved CR subsequently underwent consolidation chemotherapy with Ara‐C and etoposide (EA). WBRT was eliminated, and the estimated 2‐year OS rate was 70% in that study. Another prospective cooperative group study (NRG Oncology RTOG 0227) by Glass et al further demonstrated that RMT was safe and effective for untreated PCNSL.^34^ Although RTOG 0227 showed an ORR of 85.7% and a 2‐year OS of 80.8%,^34^ grade 3‐4 toxicities were more common with the induction therapy (66%) of RTOG 0227 than with that of CALGB 50202. In our study, RMT yielded a comparable 2‐year OS of 82.3% and a satisfactory ORR of 92.7%. More importantly, RMT tolerance was good in our study. Grade 3‐4 hematological toxicities occurred in 34.4% of the patients, and only three patients (9.4%) developed FN. In addition, our study explored the role of rituximab in PCNSL by directly comparing RMT and MT. To minimize bias, our study focused on DLBCL, and all other subtypes were excluded. The results showed that both the ORR and survival rate were significantly improved by the RMT regimen under the condition that the median follow‐up time of RMT was similar to that of MT (13.7 months vs 15.5 months, *P *= 0.797).

In our study, a total of 22 patients who responded to treatment underwent consolidation therapy. WBRT was performed in 12 patients on RMT and in 8 patients on MT. However, WBRT was not found to improve survival in either group. Only two patients on RMT underwent ASCT, and the effect could not be adequately evaluated. In the RTOG 0227 study, most of the patients underwent hyperfractionated WBRT as consolidation therapy and TMZ as maintenance therapy.^34^ In the CALGB 50202 study, patients who achieved CR after introduction therapy underwent EA as consolidation treatment, achieving a comparable survival rate to that of WBRT.^33^ Currently, WBRT is regarded as an optimal consolidation treatment for responsive PCNSL patients, but long‐term neurotoxicity should be considered.^7^, ^35^, ^36^ In addition, ASCT is considered one of the most relevant alternatives to WBRT, with increasing evidence supporting its use in PCNSL.^30^, ^37^ Randomized studies should be conducted to determine the optimal consolidation approach after RMT. Notably, a relatively high percentage of patients (56.3%) on RMT did not undergo consolidation therapy with WBRT or ASCT, but both PFS and OS were favorable. From this perspective, we suggest that RMT may be a feasible regimen in patients who cannot tolerate consolidation therapy. Moreover, a head‐to‐head trial comparing RMT and MT without consolidation therapy should be designed for elderly patients (>65 years) with PCNSL.

In the survival analyses, we further proved that therapeutic approach (RMT vs MT) was an independent prognostic factor for PFS and OS. Therefore, we considered that rituximab should be included in the first‐line regimens of future clinical trials for PCNSL. However, the two previously reported prognostic models, namely, the IESLG model^25^ and MSKCC model,^26^ did not exhibit prognostic utility for PFS or OS. Clearly, the two models, which were established in the pre‐rituximab era, may not have a sufficient prognostic effect for PCNSL. Recently, novel molecular biomarkers have shown promising prognostic effects and should be further validated in PCNSL.^34^

Several limitations should be acknowledged in this study. First, this study was restricted by its retrospective nature and inevitably suffered a patient selection bias. Second, the role of WBRT or ASCT could not be further clarified. Finally, data on long‐term neurotoxicity could not be evaluated or collected due to insufficient records.

## 5 CONCLUSION

In summary, we investigated two different regimens of MT and RMT in untreated PCNSL patients for the first time. Given its outstanding efficacy and favorable toxicity, we consider RMT to be a feasible and safe therapeutic approach as a first‐line treatment for PCNSL. Moreover, RMT is an ideal regimen for elderly patients and frail populations who may not tolerate WBRT or ASCT. Future prospective studies with large sample sizes are warranted to further validate the effect and toxicity of RMT in PCNSL.

## CONFLICT OF INTEREST

None declared.
